# The dual lipid desaturase/hydroxylase DEGS2 controls phytoceramide levels necessary to counter intestinal inflammation

**DOI:** 10.1242/dmm.050043

**Published:** 2023-09-08

**Authors:** Ran Song, Aaron Fond, Xiaohong Li, Miao Tang, Xiaoming Zhan, Ruth Gordillo, Eva Marie Y. Moresco, Bruce Beutler, Emre E. Turer

**Affiliations:** ^1^ Center for the Genetics of Host Defense, University of Texas Southwestern Medical Center, Dallas, TX 75390-8505, USA; ^2^ Department of Internal Medicine, Division of Gastroenterology, University of Texas Southwestern Medical Center, Dallas, TX 75390-8505, USA; ^3^ Department of Internal Medicine, Touchstone Diabetes Center, University of Texas Southwestern Medical Center, Dallas, TX 75390, USA

**Keywords:** Dextran sodium sulfate, N-ethyl-N-nitrosourea, Inflammatory bowel disease

## Abstract

Intestinal immunity is dependent on barrier function to maintain quiescence. The mechanisms for the maintenance of this barrier are not fully understood. Delta 4-desaturase, sphingolipid 2 (DEGS2) is a lipid desaturase and hydroxylase that catalyzes the synthesis of ceramide and phytoceramide from dihydroceramide. Using a forward genetic approach, we found and validated a mutation in *Degs2* as causative of increasing susceptibility to colitis and altering the phytoceramide balance in the colon. DEGS2 is expressed in the intestinal epithelium, and the colitis phenotype is dependent on the non-hematopoietic compartment of the mouse. In the absence of DEGS2, the colon lacks phytoceramides and accumulates large amounts of the precursor lipid dihydroceramide. In response to dextran sodium sulfate (DSS)-induced colitis, colonic epithelial cells in DEGS2-deficient mice had increased cell death and decreased proliferation compared to those in wild-type mice. These findings demonstrate that DEGS2 is needed to maintain epithelial integrity, protect against DSS-induced colitis and maintain lipid balance *in vivo*.

## Teaser

**Editor's choice:** The dual lipid desaturase/hydroxylase DEGS2 contributes to mouse intestinal homeostasis and ameliorates colitis by converting dihydroceramides to phytoceramides. Without this enzyme, intestinal epithelial cells have diminished regenerative capacity.

## INTRODUCTION

Intestinal immunity is dependent on the proper maintenance and function of an intestinal barrier. The human intestinal lumen contains an estimated 10^15^ bacteria that are maintained with minimal penetration past the mucosal surface (Johansson et al., 2008). Compromise of the intestinal barrier is, in some cases, a cause of chronic conditions, such as inflammatory bowel disease. A major question in the field is how barrier function is maintained, preventing a response by luminal microbes and consequent chronic inflammation. Intestinal barrier function requires numerous factors including vesicle trafficking, protein secretion, immune signaling, epithelial cell integrity and cell proliferation.

Delta 4-desaturase, sphingolipid 2 (DEGS2) is a lipid desaturase specific for dihydroceramide, catalyzing the formation of ceramide. DEGS2 also functions as a dihydroceramide hydroxylase, catalyzing the formation of phytoceramide via a C4 hydroxylase function (Omae et al., 2004). This differs from delta 4-desaturase, sphingolipid 1 (DEGS1), which is only able to catalyze the formation of ceramide. DEGS1, however, is thought to be the major desaturase *in vivo* for the formation of ceramide, owing to its broader expression pattern and the early lethality observed in *Degs1^−/−^* animals (Holland et al., 2007). The role of DEGS2 in the physiology of the murine intestine and intestinal homeostasis has not been explored.

To identify proteins with non-redundant function in the homeostasis of the gastrointestinal tract, we used a forward genetic screen of N-ethyl-N-nitrosourea (ENU) mutagenized mice and determined that a mutation in *Degs2* confers hypersensitivity to dextran sodium sulfate (DSS). In DEGS2-deficient mice, the intestinal epithelium fails to proliferate in response to damage from DSS. Moreover, *Degs2^−/−^* animals have perturbed intestinal lipid production under steady-state conditions. In this study, we report that DEGS2 is necessary for restoration of intestinal homeostasis and provide a phenotypic characterization of the colitis phenotype caused by loss of DEGS2 function.

## RESULTS

### Recessive mutations in *Degs2* lead to colitis susceptibility

**Fig. 1.**
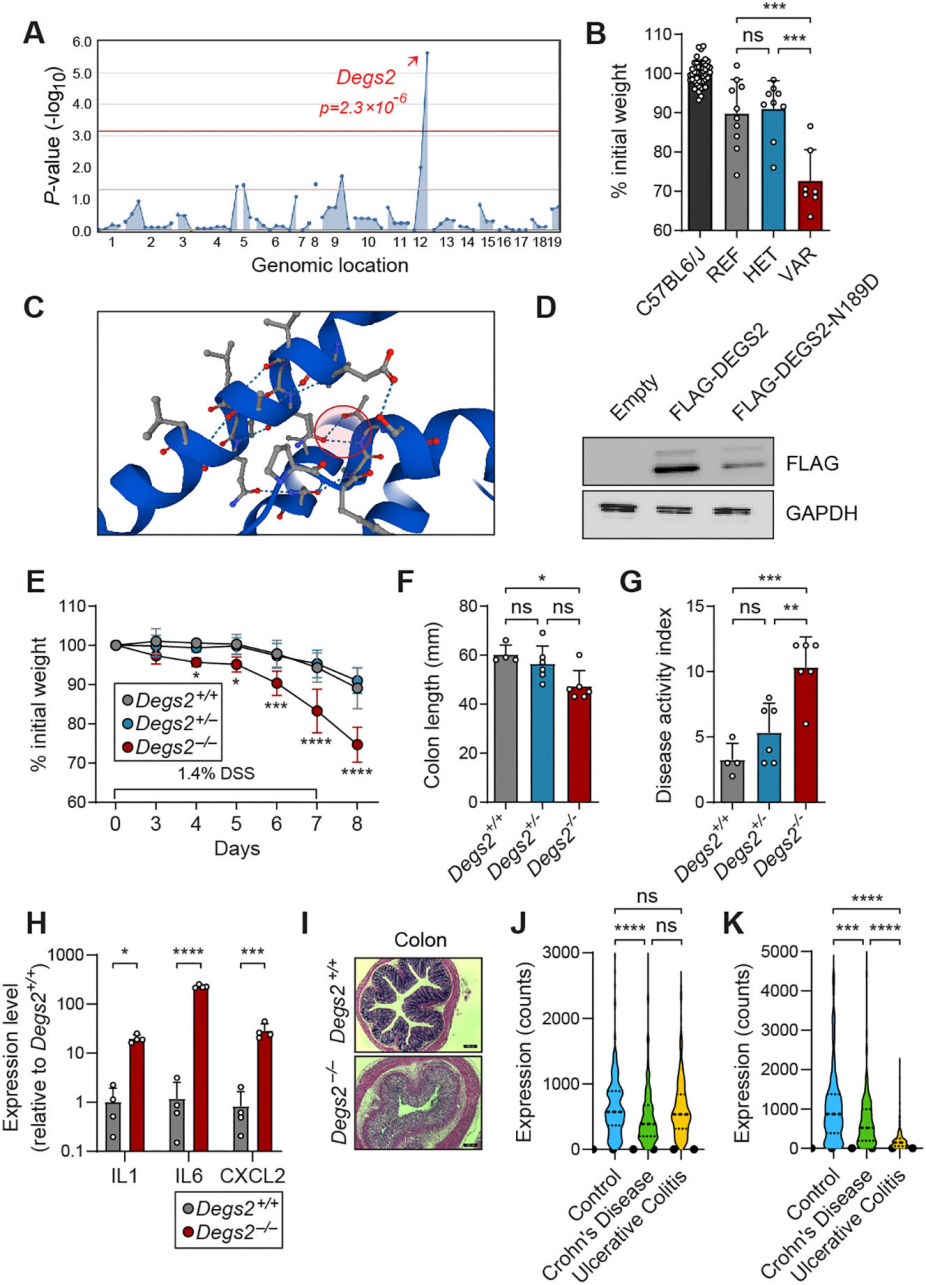
**Mapping and validation of *Degs2* mutation in the *largo* phenotype.** (A) Manhattan plot showing *P*-values of association between the dextran sodium sulfate (DSS)-induced weight loss phenotype, *largo*, and mutations identified in the *largo* pedigree, calculated using a recessive model of inheritance. The −log_10_ *P*-values (*y*-axis) are plotted versus the chromosomal positions of the mutations in the pedigree (*x*-axis). Horizontal red and pink lines represent thresholds of *P*=0.05 with or without Bonferroni correction, respectively. *P*-values for linkage of mutation in *Degs2* to the *largo* DSS phenotype are indicated. (B) Percentage of body weight loss on day 10 of DSS treatment plotted per genotype [reference (REF) *Degs2^+/+^*, *n*=9; heterozygous (HET) *Degs2^largo/+^*, *n*=9; variant (VAR) *Degs2^largo/largo^*, *n*=6]. (C) AlphaFold structure prediction for DEGS2 protein, with the N189 residue and interhelix bridge highlighted by a red circle. (D) FLAG immunoblot of MC38 cells transduced with empty vector, FLAG-tagged DEGS2-mCherry or FLAG-tagged-N189D-mCherry. MC38 cells were sorted for mCherry positivity. (E) Weight loss analysis of CRISPR/Cas9-targeted *Degs2^+/+^*, *Degs2^+/−^* and *Degs2^−/−^* mice after 1.4% DSS treatment (*n*=5 for all groups, representative of at least three experiments). (F,G) Colon length (F) and disease activity index (G) of mice after 7 days of DSS challenge. (H) Quantitative PCR analysis of colon tissue from DSS-treated *Degs2^+/+^* and *Degs2^−/−^* mice (*n*=4 mice per genotype). (I) Representative Hematoxylin and Eosin staining of *Degs2^+/+^* and *Degs2^−/−^* colons after 7 days of DSS treatment. All experiments were repeated at least three times. Scale bars: 100 μm. (J,K) *DEGS2* expression in small intestine (J) and colon (K) biopsies of inflammatory bowel disease patients as determined by meta-analysis of RNA-sequencing datasets (Massimino et al., 2021). Data are expressed as means±s.d., and significance was determined by two-way ANOVA with Sidak's multiple comparisons (E,H) or one-way ANOVA with multiple comparisons (B,F,G,J,K) (ns, not significant; *P<0.05, **P<0.005, ***P<0.001, ****P<0.0001).

Random mutations were generated using ENU, and a previously described inbreeding scheme was employed to produce mice in the heterozygous and homozygous mutant state (McAlpine et al., 2018; Song et al., 2023; Turer et al., 2017; Wang et al., 2019, 2015). We subjected 55,867 third-generation (G3) mice from 2039 pedigrees to 1.4% DSS in their drinking water, and body weights of the mice were recorded after 7 and 10 days of DSS treatment. The resulting screen led to the mapping of 119 candidate genes with phenovariance to DSS-induced colitis. A DSS susceptibility phenotype in a single pedigree (R0044) was designated as *largo* and mapped to a mutation on chromosome 12: *Degs2* with a recessive model of inheritance (*P*=2.3×10^−6^) (Fig. 1A,B). The *Degs2* mutation in this pedigree was a missense mutation (N189D) and predicted to be probably damaging by PolyPhen-2 (Adzhubei et al., 2010), with a score of 1.000. AlphaFold structure analysis indicated that the mutated residue (Asn189) is present in an alpha helix, which forms a hydrogen bond from Asn189 to Thr122 on a neighboring alpha helix, indicating that it might be necessary for DEGS2 conformational stability (Fig. 1C) (Jumper et al., 2021; Varadi et al., 2022). Lentivirally transduced MC38 colorectal cancer cells, sorted on an mCherry reporter, demonstrated decreased expression of the mutant (N189D) compared to wild-type DEGS2 (Fig. 1D). These data indicate that a destabilizing mutation in *Degs2* increases susceptibility to DSS-induced colitis.

To verify that *Degs2* was the causative gene in this pedigree, we generated mice with an independent CRISPR/Cas9 targeted mutation, a 14 bp frameshift allele of the *Degs2* gene. The CRISPR/Cas9-targeted mice (*Degs2^−/−^*) were challenged with DSS and showed significantly increased colitis, as assessed by weight loss, disease activity index (a composite score of weight loss, rectal bleeding and diarrhea) and colonic shortening (Fig. 1E-G)**.** By day 6 of DSS treatment, the expression of proinflammatory genes (*Il6*, *Cxcl2* and *Il1b*) was markedly increased in the distal colons of *Degs2^−/−^* mice compared to those of *Degs2^+/+^* mice (Fig. 1H). Colons from the DSS-treated *Degs2^−/−^* mice showed marked histopathological changes characterized by infiltration of lymphocytes and loss of crypt architecture (Fig. 1I). No histopathological difference was noted in the small intestines of these animals after DSS treatment (Fig. S1). This indicates that the *Degs2* mutation is causative of the *largo* phenotype and impairs intestinal homeostasis *in vivo*.

Analysis of RNA-sequencing data from the IBD Transcriptome and Metatranscriptome Meta-Analysis (TaMMA) framework (Massimino et al., 2021) revealed that, in human inflammatory bowel disease patients, *DEGS2* expression was decreased in colonic and small intestinal biopsies (Massimino et al., 2021). This decrease was most observable in the colons of ulcerative colitis patients; a smaller decrease was observed in the small intestine and colons of Crohn's disease patients (Fig. 1J,K). These data indicate that DEGS2 plays a role in human intestinal homeostasis and the pathogenesis of inflammatory bowel disease.

### DEGS2 controls sphingolipid balance in the intestine

**Fig. 2.**
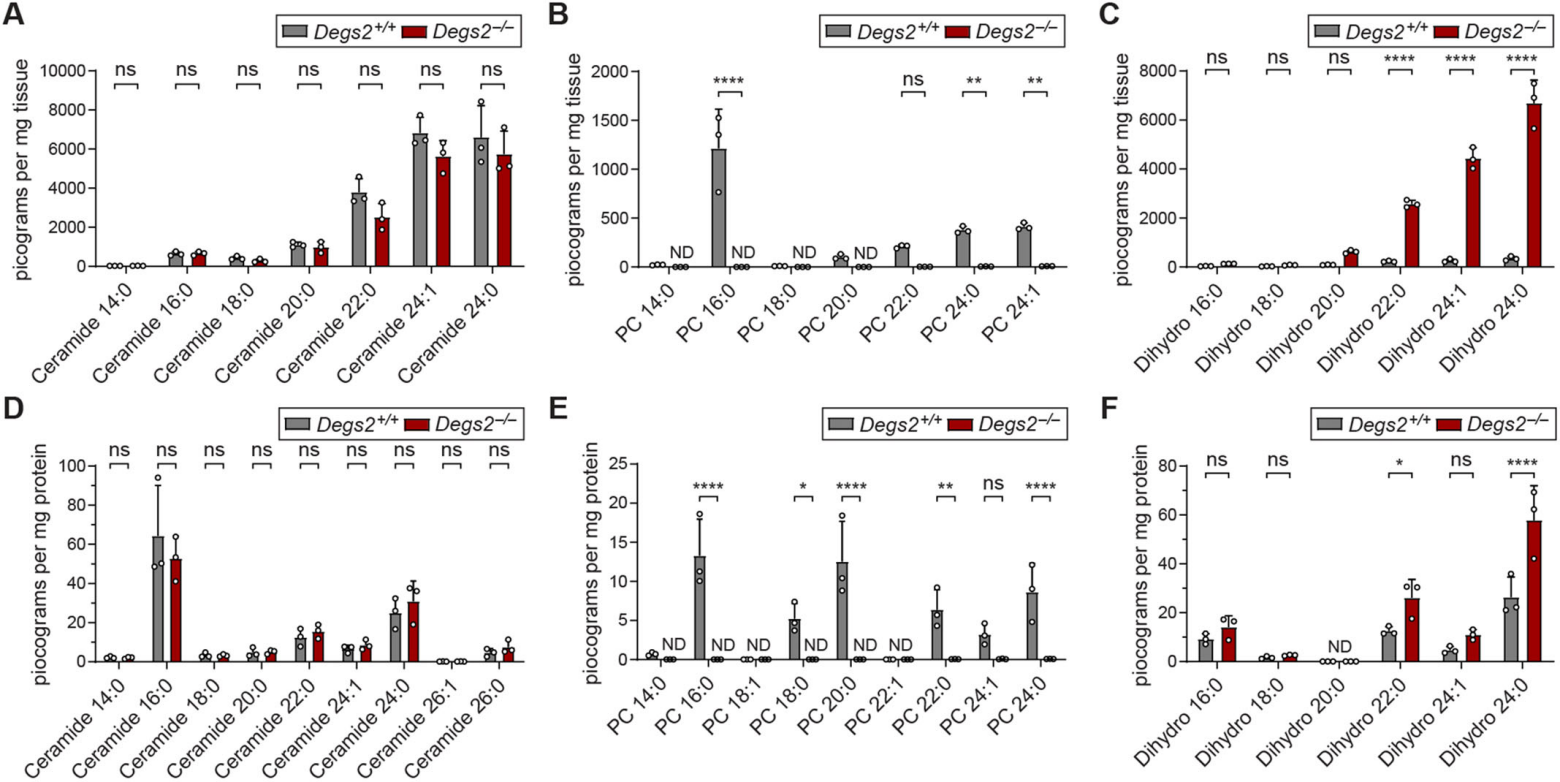
**Quantitation of intestinal ceramide levels.** (A-C) Determination of ceramide (A), phytoceramide (PC; B) and dihydroceramide levels (Dihydro; C) in colon tissues of *Degs2^+/+^* (*n*=3) and *Degs2^−/−^* (*n*=3) mice. Data are normalized to tissue weight. (D-F) Quantification of ceramide (D), PC (E) and Dihydro (F) levels from small intestinal enteroids derived from *Degs2^+/+^* (*n*=3) and *Degs2^−/−^* (*n*=3) mice. Lipid chain length and number of desaturations are indicated in the figure. Data are expressed as means±s.d., and significance was determined by two-way ANOVA with Sidak’s multiple comparisons (ns, not significant; *P<0.05, **P<0.005, ****P<0.0001). Data are representative of at least three independent experiments. ND, not detected.

DEGS2 has been shown *in vitro* to act as a lipid desaturase and hydroxylase specific for converting dihydroceramide to ceramide or phytoceramide, respectively. To understand the effects of DEGS2 *in vivo*, we isolated colons from *Degs2^+/+^* and *Degs2^−/−^* animals and analyzed their sphingolipid profiles by liquid chromatography–electrospray ionization–tandem mass spectrometry (LC-MS/MS). DEGS2-deficient colons displayed a striking loss of phytoceramides but normal levels of ceramides (Fig. 2A,B). In addition to loss of phytoceramide, colons from mutant animals were noted to have a dramatic increase in the precursor sphingolipids, dihydroceramides (Fig. 2C). To demonstrate that these effects on sphingolipids are epithelial intrinsic, the sphingolipid profiles of *Degs2^+/+^* and *Degs2^−/−^* enteroids were determined. As in whole tissue, loss of phytoceramides was noted (Fig. 2E), with a commensurate increase in dihydroceramides (Fig. 2F); ceramides were present at normal levels (Fig. 2D). These data demonstrate that DEGS2 is necessary *in vivo* for the clearance of ceramide precursors and the production of phytoceramides in the mammalian intestine.

### *Degs2* mutant mice have normal intestinal differentiation and permeability

**Fig. 3.**
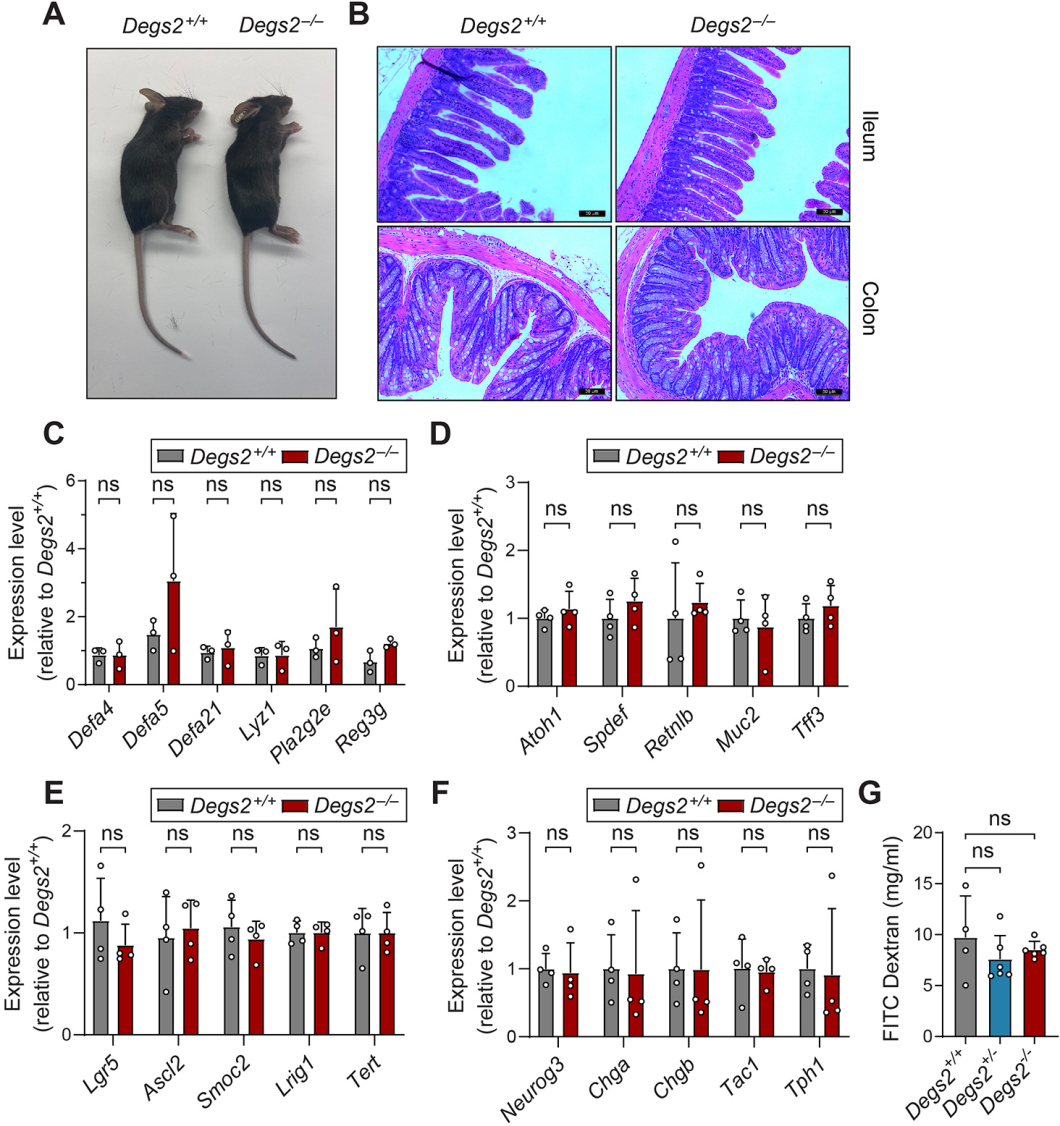
**Normal intestinal differentiation and permeability.** (A) Representative 10-week-old female mice. (B) Representative Hematoxylin and Eosin staining of ilea and colons of *Degs2^+/+^* and *Degs2^−/−^* mice. Scale bars: 50 μm. (C,D) Quantitative PCR of Paneth and goblet cell marker genes from ileum (C) and colon (D) (four mice per genotype). (E,F) RNA sequencing of stem cell (E) and transit amplifying cell marker (F) genes from distal colonic epithelium of *Degs2*^+/+^ and *Degs2*^−/−^ mice. (G) Serum fluorescence after gavage of FITC-dextran in *Degs2^+/+^* (*n*=4), *Degs2^+/−^* (*n*=6) and *Degs2^−/−^* (*n*=7) mice. Data are expressed as means±s.d., and no statistically significant differences were found between genotypes by two-way ANOVA (ns, not significant). Data are representative of at least three independent experiments.

*Degs2* expression is dependent on the transcription factor aryl hydrocarbon receptor nuclear transporter (ARNT), and mice with skin-specific deletion of ARNT die prematurely owing to increased skin permeability, attributed to loss of *Degs2* and disrupted ceramide composition in the skin (Takagi et al., 2003). Notably, no gross defect in skin or hair loss was observed in *Degs2^−/−^* mice (Fig. 3A). No histological differences were noted between *Degs2^+/+^* and *Degs2^−/−^* small intestine and colonic tissue prior to DSS treatment (Fig. 3B). There were also no differences in expression of Paneth cell-specific and goblet cell-specific genes as measured by quantitative PCR, indicating that differentiation of Paneth and goblet cells is grossly intact in *Degs2* mutant mice (Fig. 3C,D). RNA sequencing of distal colonic epithelium did not reveal any differences in stem cell or transit amplifying cell marker genes (Fig. 3E,F). To assess intestinal permeability, mice were orally gavaged with fluorescein isothiocyanate (FITC)-dextran, and the ability of dextran to cross the mucosal barrier was evaluated by fluorescence intensity in venous blood after 4 h. No difference in fluorescence was seen between *Degs2^+/+^*, *Degs2^+/−^* and *Degs2^−/−^* animals (Fig. 3G). Overall, these results demonstrate that colitis susceptibility in the *Degs2^−/−^* mice is not due to increased baseline intestinal permeability or altered cell differentiation.

### Hematopoietic extrinsic control of colitis

**Fig. 4.**
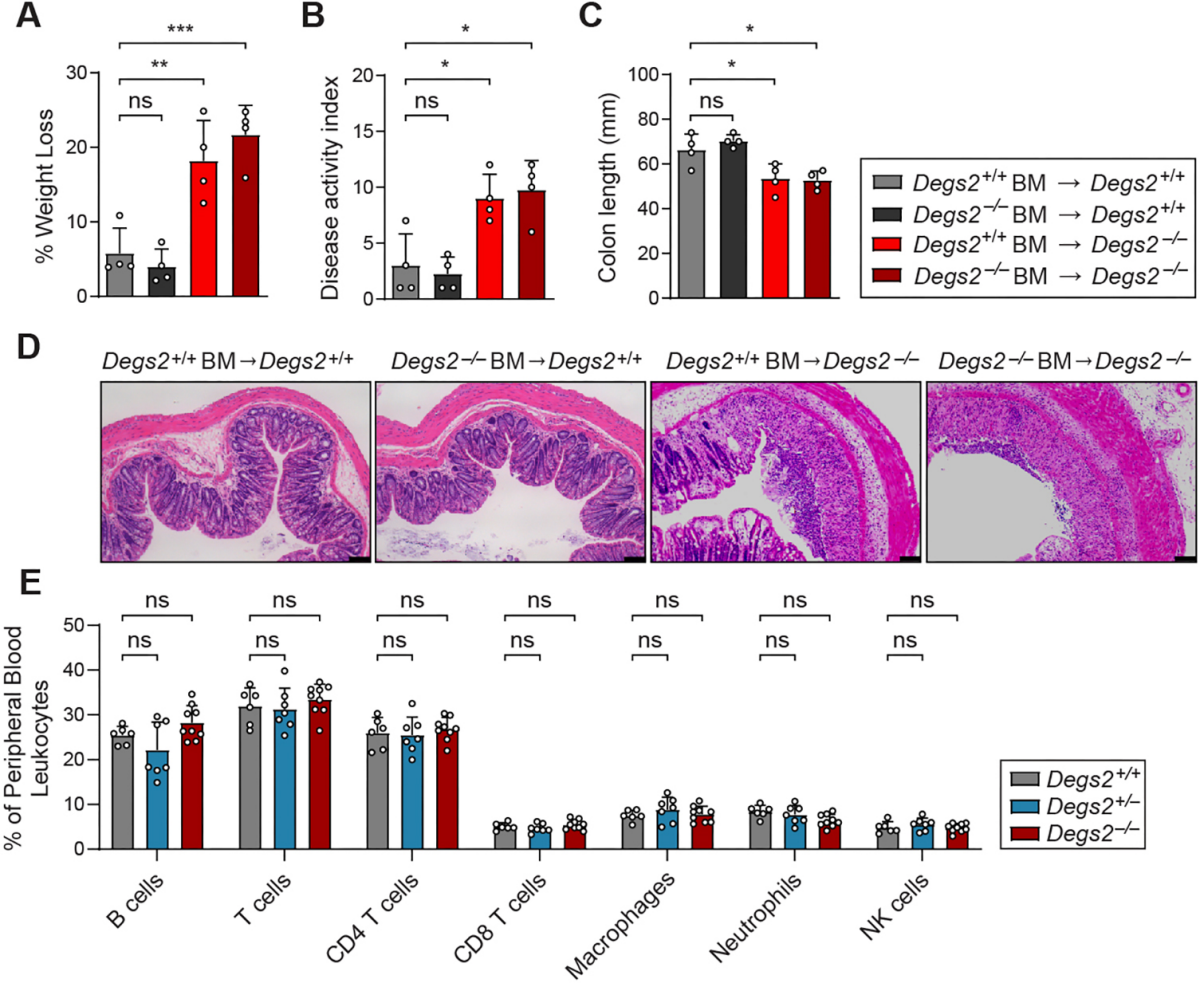
**Hematopoietic extrinsic role of DEGS2 in murine colitis.** (A-C) Bone marrow chimeras were generated, and percentage of initial body weight (A), disease activity index (B) and colon length (C) were determined after 8 days of 1.4% DSS challenge (*n*=4 for all groups). (D) Representative Hematoxylin and Eosin staining of colons of bone marrow (BM) chimeras after 8 days of DSS treatment. Scale bars: 50 μm. (E) Flow cytometry analysis of B cells (B220^+^), T cells (CD3^+^), CD4^+^ T cells, CD8^+^ T cells, macrophages (F4/80^+^), neutrophils (CD11B^+^ F4/80^−^) and natural killer (NK) cells (NK1.1^+^ CD3^−^) in *Degs2^+/+^* (*n*=6), *Degs2^+/−^* (*n*=6) and *Degs2^−/−^* (*n*=9) mice. Data are expressed as means±s.d., and significance was determined by one-way ANOVA with multiple comparisons (A-C) and two-way ANOVA (E) (ns, not significant; *P<0.05, **P<0.005, ***P<0.0005).

*Degs2* mRNA is most highly expressed in the kidney, skin and intestinal epithelium, especially in the crypt (Omae et al., 2004). To probe the contribution of the hematopoietic compartment to the colitis phenotype, we generated bone marrow chimeric mice. Chimeric wild-type recipient mice with *Degs2^−/−^* hematopoietic cells did not exhibit increased susceptibility to DSS challenge compared to that of control CD45.1 mice receiving wild-type bone marrow (Fig. 4A). Chimeric *Degs2^−/−^* recipient mice, irrespective of donor bone marrow, were not protected from DSS challenge and lost an average of 20% of their initial body weight by day 8 (Fig. 4A). The weight loss coincided with increased disease activity index (Fig. 4B) as well as reduced colon length (Fig. 4C). Histologic examination of distal colons of *Degs2^−/−^* recipient mice displayed marked increases in inflammatory infiltrates compared with those of *Degs2^+/+^* recipients (Fig. 4D). Consistent with these data, the peripheral immune profile of DEGS2-deficient animals demonstrated no differences in the frequencies of major immune subsets compared to those of wild-type and heterozygous littermates (Fig. 4E). Taken together, these findings indicate that the increased sensitivity of *Degs2^−/−^* mice is caused by DEGS2 deficiency in non-hematopoietic cells.

### Colonocytes from *Degs2^−/−^* mice have decreased regenerative capacity

**Fig. 5.**
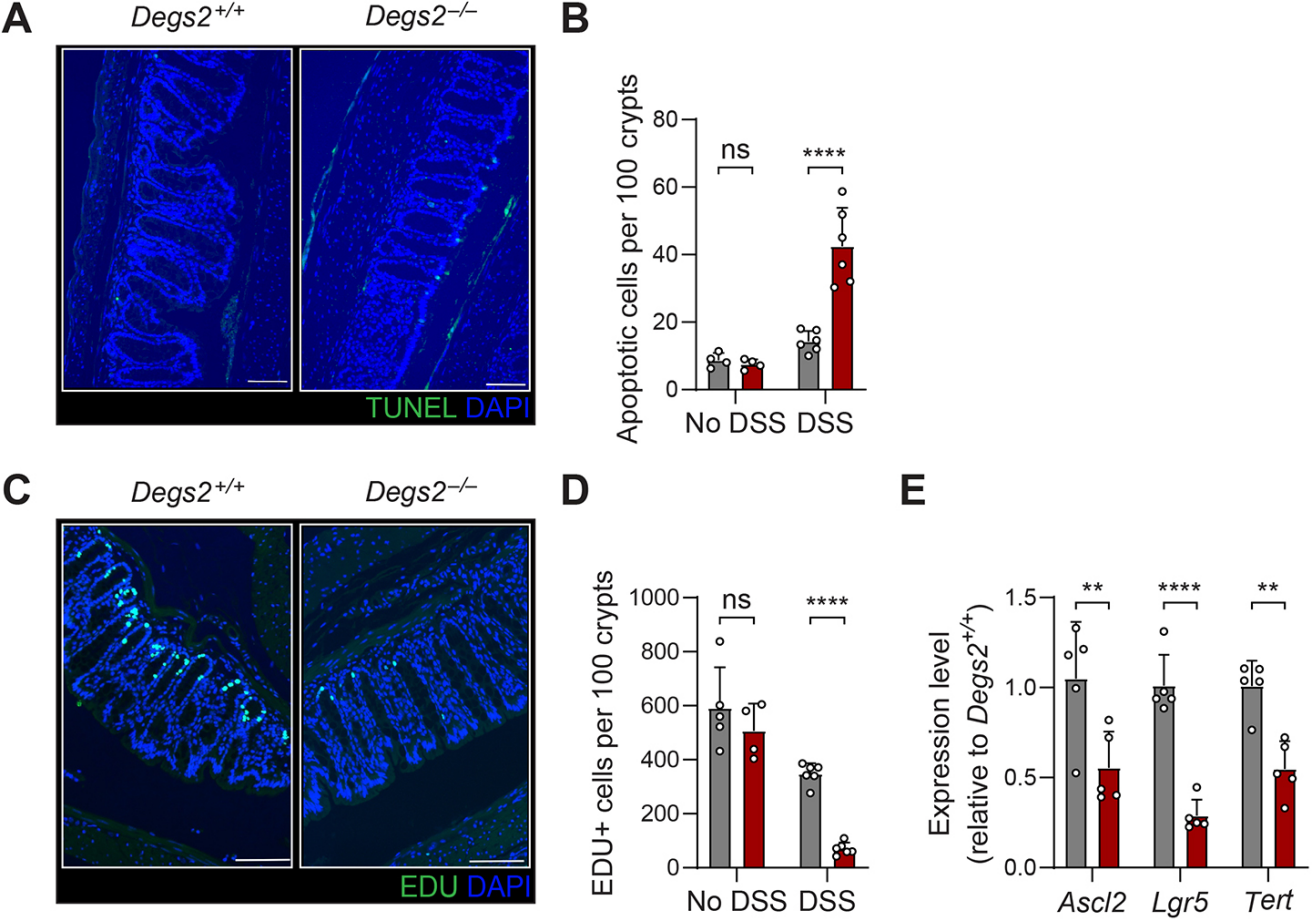
**Decreased regenerative capacity of DEGS2-deficient intestinal epithelium.** (A) Representative images of staining in *Degs2^+/+^* and *Degs2^−/−^* distal colons after 4 days of DSS treatment. Scale bars: 100 μm. (B) Quantitation of TUNEL-labeled cells from at least 50 crypts of *Degs2^+/+^* and *Degs2^−/−^* distal colons. (C) Representative images of EDU labeling in *Degs2^+/+^* and *Degs2^−/−^* distal colons after 4 days of DSS treatment. Scale bars: 100 μm. (D) Quantitation of EDU-labeled cells from at least 50 crypts of *Degs2^+/+^* and *Degs2^−/−^* distal colons. DAPI was used as a counterstain. (E) Quantitative PCR analysis of colon tissues from DSS-treated *Degs2^+/+^* and *Degs2^−/−^* mice (*n*=5 per genotype) analyzing intestinal stem cell markers. Data are expressed as means±s.d., and significance was determined by two-way ANOVA with Sidak's multiple comparisons (B,D) or unpaired two-tailed Student *t*-test (E) (ns, not significant; **P<0.005, ****P<0.0001).

Ceramide species are known to have pleiotropic effects on cells, altering cell death pathways as well as the cell cycle. Examination of the distal colons from *Degs2^−/−^* mice after 4 days of DSS treatment displayed a statistically significant twofold increase in terminal deoxynucleotidyl transferase dUTP nick-end labeling (TUNEL)-positive cells per crypt, indicating an increase in cell death at this early time point (Fig. 5A,B). Additionally, using EDU labeling, we found that proliferation in the colon was significantly decreased in mutant mice compared to that in wild-type mice after DSS treatment (Fig. 5C,D). Notably, a decrease in stem cell markers was observed in the colons of *Degs2^−/−^* mice after DSS treatment (Fig. 5E). This defect in stem cell markers was not observed in untreated mice (Fig. 3E). These data indicate that DEGS2 deficiency affects the proliferation and cell death of the intestinal epithelium, consistent with a defect in regenerative response to stress.

## DISCUSSION

Using forward genetics, we demonstrated that a damaging mutation in *Degs2* renders mice susceptible to DSS-induced colitis, which we validated in an independent CRISPR/Cas9-targeted *Degs2* mutant strain with a clean genetic background (C57BL/6J). In the colon, DEGS2 appears to regulate predominantly the balance of phytoceramide and dihydroceramide, whereas ceramide levels are not affected by DEGS2 deficiency. The altered phytoceramide/dihydroceramide levels lead to increased cell death and a defect in proliferation in response to acute colitis.

The effect of DEGS2 deletion in increasing susceptibility to DSS-induced colitis in mice with intact DEGS1 function indicates that DEGS2 has evolved a non-redundant function that cannot be performed by DEGS1. This could be due to a difference in cellular expression patterns or the additional hydroxylase activity of DEGS2. Recent single-cell RNA sequencing indicates *DEGS2* expression prominently in early colonic epithelial progenitors (e.g. stem cells and transit amplifying cells) of human patients with little expression of *DEGS1* in these cells (Smillie et al., 2019). Interestingly, ceramide-dihydroceramide balance has also been implicated in hematopoietic stem cell renewal via a proteostasis/autophagy program not observed in more differentiated cells (Xie et al., 2019). We find that DEGS2-deficient colons have decreased expression of stem cell markers after stress. These data raise an interesting question of whether DEGS2 is needed for intestinal stem cell homeostasis and renewal in response to stress and is an area for future investigation.

Changes in sphingolipid balance have been shown to have pleiotropic effects on cells, altering cell death and proliferation pathways (Lachkar et al., 2021). A recent report showed that elevations in dihydroceramides in *Drosophila* cause epithelial cell degeneration via an increase in reactive oxygen species (Jung et al., 2017). An attractive hypothesis, therefore, is that dihydroceramides elevated as a result of DEGS2 deficiency play a similar role in the mammalian intestine, leading to increased reactive oxygen species and epithelial degeneration.

DEGS2 and phytoceramide levels have previously been shown to correlate with intestinal disease. Using the IBD TaMMA (Massimino et al. 2021), we see *DEGS2* mRNA downregulated in Crohn's disease and ulcerative colitis biopsy samples. Human DEGS2 has also been found to be increased in colorectal tumors and necessary for the proliferation of cell lines *in vitro* (Guo et al., 2021). In mice treated with 2,4,6-trinitrobenzene sulphonic acid (TNBS), another inducer of acute colitis, there is a decrease in intestinal epithelial *Degs2* levels and phytosphingosine (Montenegro-Burke et al., 2021). Because phytoceramide is a precursor to phytosphingosine, this finding is in concordance with our findings that lack of DEGS2, and subsequent absence of phytoceramides, in the mouse gut increases susceptibility to colitis.

Our current study is limited to acute chemical colitis using DSS as a model system. DSS is a well-appreciated model of intestinal damage and regeneration but might not fully recapitulate human inflammatory bowel disease. Given the observed difference in human samples, it will be of interest to determine whether DEGS2 is necessary in immune-mediated models, such as IL10 deficiency or CD4^+^ T-cell transfer models. The use of these models would broaden the significance of DEGS2 in epithelial cell regeneration, which is a critical determinant in human disease.

In summary, we have identified an essential and non-redundant role for DEGS2 in epithelial cell barrier function and intestinal homeostasis *in vivo*. The present study highlights the importance of sphingolipid regulation for epithelial cell integrity. Future work will focus on determining the intracellular events controlled by DEGS2 in the intestinal epithelium.

## MATERIALS AND METHODS

### Mice

Eight- to ten-week-old C57BL/6J mice were purchased from The Jackson Laboratory. ENU mutagenesis was performed as previously described (Wang et al., 2015). For the DSS-induced colitis induction, mice received 1.4% (w/v) DSS in the drinking water for 7 days followed by 3 days off DSS. Body weight was recorded daily and reported as the amount of weight loss from the pre-treatment weight. Disease activity index score is a composite score of weight loss, stool bleeding and stool consistency determined as previously described (Kim et al., 2012) [weight loss: 0 (no loss), 1 (1-10% loss of body weight), 2 (10-15% loss of body weight), 3 (15-20% loss of body weight), 4 (>20% loss of body weight); stool consistency: 0 (normal), 2 (loose stool), 4 (diarrhea); and bleeding: 0 (no blood), 2 (hemoccult positive), 4 (gross bleeding and/or blood around anus)]. All mice were housed in the University of Texas (UT) Southwestern vivarium. All procedures performed were performed in accordance with institutionally approved protocols.

### Generation of the *Degs2*^−/−^ mouse strain using the CRISPR/Cas9 system

To generate the *Degs2^−/−^* mouse strain, female C57BL/6J mice were superovulated by injection of 6.5 U pregnant mare serum gonadotropin (Millipore), followed by injection of 6.5 U human chorionic gonadotropin (Sigma-Aldrich) 48 h later. The superovulated mice were subsequently mated overnight with C57BL/6J male mice. The following day, fertilized eggs were collected from the oviducts, and *in vitro*-transcribed Cas9 mRNA (50 ng/μl) and *Degs2* small-base-pair guide RNA (50 ng/μl; 5′-ttaatacgactcactatagGAGTCCCCGTACTAACCAGC-3′) were injected into the cytoplasm or pronucleus of the embryos. The injected embryos were cultured in M16 medium (Sigma-Aldrich) at 37°C in 5% CO_2_. For the production of mutant mice, two-cell-stage embryos were transferred into the ampulla of the oviduct (10-20 embryos per oviduct) of pseudo-pregnant Hsd:ICR (CD-1) female mice (Harlan Laboratories).

### Generation of bone marrow chimeric mice

Recipient mice were lethally irradiated with two 7-Gy exposures to X-irradiation administered 5 h apart. Femurs derived from donor *Degs2^+/+^* or *Degs2^−/−^* mice were flushed with phosphate-buffered saline (PBS) using a 25G needle. The cells were centrifuged at 700 ***g*** for 5 min, and cells were resuspended in 1 ml PBS, transferred into 1.5-ml Eppendorf tubes and kept on ice. Bone marrow cells from *Degs2^+/+^* or *Degs2^−/−^* mice were transferred into the indicated recipient mice through intravenous injection. For 4 weeks after injection, mice were maintained on antibiotics. Analysis of DSS colitis susceptibility was performed 10 weeks after irradiation and reconstitution.

### Cell culture, transfection and infection

The Lenti-X 293T cells were grown at 37°C in Dulbecco's modified Eagle medium (DMEM; Sigma-Aldrich)/10% (v/v) fetal bovine serum (FBS; Gibco)/1% antibiotics (Life Technologies) in 5% CO_2_. Transfection of empty vector (pLVX-mCherry) or plasmids containing *Degs2* (pLVX-mDEGS2-mCherry) or mutant *Degs2* (pLVX-N189D-mCherry) was carried out using Lipofectamine 2000 (Life Technologies) according to the manufacturer's instructions. Cells were harvested between 36 and 48 h post-transfection. The MC38 cells were grown at 37°C in DMEM (Life Technologies)/10% (v/v) FBS (Gibco)/1% antibiotics (Life Technologies) in 5% CO_2_. All cell lines were regularly tested for mycoplasma contamination.

### Histology and immunostaining

Freshly isolated colons and small intestines were fixed in formalin and embedded in paraffin. Hematoxylin and Eosin staining was conducted using a standard protocol by the UT Southwestern Histology core.

### Small intestinal enteroid culture

Small intestinal enteroids were derived as previously described (Sato et al., 2009). In brief, small intestinal epithelial crypt fractions were isolated using 2 mM EDTA. A total of five epithelial fractions were isolated per genotype and crypt number, and purity was assessed through light microscopy. A total of 200-500 crypts were plated in 40 μl Matrigel (Corning) added to a 24-well dish. Cells were incubated in IntestiCult medium (StemCell Technologies) until harvesting for sphingolipid concentration determination.

### FITC-dextran permeability assay

Intestinal permeability was assessed by administration of FITC-dextran 4000 (Sigma-Aldrich). After a 4-h fast, mice were orally gavaged with FITC-dextran (20 mg/100 g body weight). Whole blood was obtained from the submandibular plexus, and fluorescence was measured in the serum by a fluorometer (BioTek) at 488 nm.

### Quantitative reverse transcription PCR

Total RNA from colon was isolated using TRIzol reagent (Thermo Fisher Scientific) as per the manufacturer's instructions. The isolated RNA was further purified in a silica column (Invitrogen) to remove any excess DSS. One microgram of RNA was reverse transcribed to cDNA with SuperScript III First-Strand Synthesis System for RT-PCR (Life Technologies). Transcript levels were analyzed using iTaq Universal SYBR Green Supermix (Bio-Rad) on a Step One Plus Real-Time PCR System (Life Technologies). Please see Table S1 for primer sets. Relative expression was calculated using the ΔΔCt standardization method using *Actb*.

### Flow cytometry

Red blood cells were lysed with hypotonic buffer (eBioscience). Samples were washed with FACS staining buffer [PBS with 1% (w/v) bovine serum albumin]. Samples were stained for 1 h at 4°C, in 100 μl of a 1:200 cocktail of fluorescence-conjugated antibodies against eight cell surface markers encompassing the major immune lineages – B220 (BD Biosciences, clone RA3-6B2, 557957; RRID:AB_396957), CD19 (BD Biosciences, clone 1D3, 563557; RRID:AB_2722495), CD3ε (BD Biosciences, clone 145-2C11, 100306; RRID:AB_312671), CD4 (BD Biosciences, clone RM4-5, 563727; RRID:AB_2728707), CD8α (BioLegend, clone 53-6.7, 100752; RRID:AB_2563057), CD11B (BioLegend, clone M1/70, 101257; RRID:AB_2565431), F4/80 (Tonbo, clone BM8.1, 50-4801; RRID:AB_2621795), NK 1.1 (BioLegend, clone OK136, 564143; RRID:AB_2738617) – and 1:200 Fc block (Tonbo, clone 2.4G2, 70-0161-M001).

### RNA-sequencing preparation and analysis

RNA was extracted using an RNeasy Mini Kit (Qiagen) according to the manufacturer's protocol. RNA quantity and purity were assessed on a NanoDrop 2000 spectrophotometer (Thermo Fisher Scientific), and integrity was measured on an Agilent Bioanalyzer 2100 (Agilent Technologies). RNA-sequencing libraries were prepared with a KAPA Stranded RNA-Seq Kit with RiboErase (HMR) (KAPA Biosystems) according to the manufacturer's protocol. For human expression datasets, RNA-sequencing data showing *DEGS2* expression were downloaded from the IBD TaMMA via Github (https://github.com/Humanitas-Danese-s-omics/ibd-meta-analysis-data). Terminal ileum and colon biopsy samples from control, Crohn's diease and ulcerative colitis cases were compared, as indicated.

### Ceramide quantitation

Sphingolipids analyses were performed at the UT Southwestern Metabolic Phenotyping Core facility. Colon frozen tissues were homogenized in cold saline solution. Total protein quantification was performed by the bicinchoninic acid assay. The remaining aqueous lysate was immediately quenched by adding 4 ml sphingolipid organic extraction solvent [isopropanol:ethyl acetate, 15:85 (v/v)]. Immediately afterward, 40 µl internal standard solution {Ceramide/Sphingoid Internal Standard Mixture II (Avanti Polar Lipids, Alabaster, AL, USA) at a tenfold dilution in methanol combined with a mixture of C16 Ceramide-d7 (d18:1-d7/16:0), C18 Ceramide-d7 (d18:1-d7/18:0), C24 Ceramide-d7 (d18:1-d7/24:0) and C24:1 Ceramide-d7 [d18:1-d7/24:1(15Z)]} was added at a concentration of 2.4 µM. The mixture was vortexed and 1.5 ml high-performance liquid chromatography (HPLC) water was added. Two-phase liquid extraction was performed, the supernatant was transferred to a new tube, and the aqueous phase was re-extracted. Supernatants were combined and split into two fractions of 2.0 ml each. The first fraction was dried under a nitrogen stream, and the dried residue was reconstituted in MeOH. Sphingolipid profiling was conducted by LC-MS/MS, using a Nexera X2 UHPLC coupled to an LCMS-8050 (Shimadzu Scientific Instruments, Columbia, MD, USA). Lipid separation was achieved by reverse-phase liquid chromatography on a 2.1×150 mm, 2.7 µm Ascentis Express C8 HPLC column (Supelco, Bellefonte, PA, USA), using a gradient elution with H_2_O, 5 mM ammonium formate, 0.8% formic acid (Holland et al., 2011; Zhang et al., 2021). The second organic fraction was dried under a nitrogen stream and reconstituted in 2.0 ml Folch's solution. Then, 150 µl of 1 M KOH solution in MeOH was added. The mixture was incubated at 37°C for 2 h, then neutralized at room temperature with 20 µl acetic acid. The resulting mixture was dried down under a nitrogen stream, and two additional two-phase liquid extraction cycles were performed to eliminate the salts originated during the hydrolysis. The final organic extract was dried down under a nitrogen stream and reconstituted in 200 µl methanol. This sample was injected for the analysis of phytoceramides. Phytoceramide profiling was conducted by LC-MS/MS, using a Nexera X2 UHPLC coupled to an LCMS-8060 (Shimadzu Scientific Instruments). Lipid separation was achieved by reverse-phase liquid chromatography on a 2.1×150 mm, 2.7 µm Ascentis Express C8 HPLC column (Supelco), using a gradient elution with H_2_O, 5 mM ammonium formate, 0.8% formic acid. Hydrolyzed yeast lipid extracts (0.6 mg/ml) were used as a reference mixture for phytoceramide species owing to their abundance in yeast. Retention times and compound identity were determined by analyzing the yeast lipid extract in a Shimadzu LCMS-9030 Q-TOF mass spectrometer. LabSolutions V 5.82 and LabSolutions Insight V 2.0 program packages (Shimadzu Scientific Instruments) were used for data processing.

### Data reproducibility and statistical analysis

Source data for Figs 1-5 are available in Dataset 1. All strains were generated and maintained on the same pure inbred background (C57BL/6J); experimental assessment of variance was not performed. No data were excluded. The investigators were aware of genotypes and group allocations during experiments. Comparisons of differences were between two unpaired experimental groups in all cases. An unpaired two-tailed *t*-test (Student's *t-*test) is appropriate and was used for such comparisons. One-way or two-way ANOVA with Sidak's multiple comparisons was applied to experiments with three or more groups. The phenotype of mice (C57BL/6J) and primary cells of these mice is expected to follow a normal distribution. The statistical significance of differences between experimental groups was determined with GraphPad Prism 7 software and unpaired two-tailed Student's *t-*test. *P*<0.05 was considered statistically significant. No pre-specified effect size was assumed, and in general four mice or more for each genotype or condition were used in experiments; this sample size was sufficient to demonstrate statistically significant differences in comparisons between two unpaired experimental groups by an unpaired two-tailed Student's *t*-test.

## Supplementary Material

10.1242/dmm.050043_sup1 Supplementary information
