# Supplementary material for: The dual lipid desaturase/hydroxylase DEGS2 controls phytoceramide levels necessary to counter intestinal inflammation
Source: Dis Model Mech. 2023 Sep 8;16(9):dmm050043. doi: 10.1242/dmm.050043 (PMC10499023; doi:10.1242/dmm.050043)
Supplement: Supplementary information [file dmm-16-050043-s1.pdf]

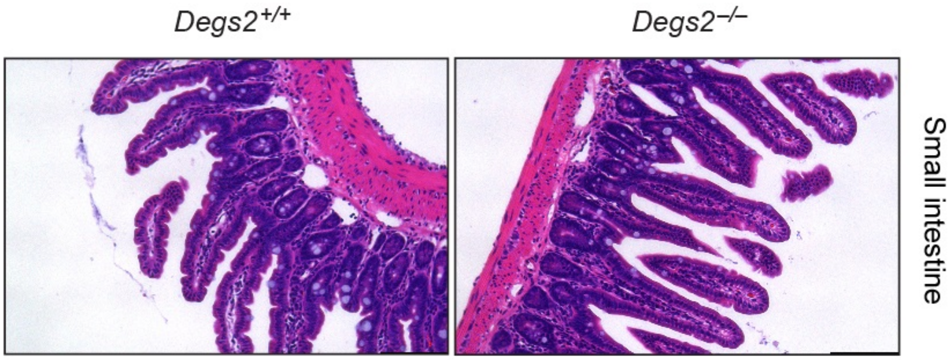

Fig. S1. Histology of terminal ileum after DSS treatment. Representative Hematoxylin and Eosin staining of *Degs2*$^{+/+}$ and *Degs2*$^{-/-}$ small intestine after DSS treatment. All experiments repeated at least three times.

Table S1. qPCR primer sets

Gene	Forward Primer	Reverse Primer
Actb	ATGGAGGGGAATACAGCCC	TTCTTTGCAGCTCCTTCGTT
Ascl2	AAGCACACCTTGACTGGTACG	AAGTGGACGTTTGCACCTTCA
Cxcl2	TCCAGGTCAGTTAGCCTTGC	CGGTCAAAAAGTTTGCCTTG
Defa-rs1	caccacccaagctccaaatacacag	atcgtgaggaccaaaagcaaatgg
Defa21	ccaggggaagatgaccaggctg	tgcagcgacgatttctacaaaggc
DEFA4	CCAGGGGAAGATGACCAGGCTG	TGCAGCGACGATTTCTACAAAGGC
DEFA5	AGGCTGATCCTATCCACAAAACAG	TGAAGAGCAGACCCTTCTTGGC
Defcr1	tcaagaggctgcaaaggaagagaac	tggtctccatgttcagcgacagc
interleukin-1	GGTCAAAGGTTTGGAAGCAG	TGTGAAATGCCACCTTTTGA
Interleukin-6	ACCAGAGGAAATTTTCAATAGGC	TGATGCACTTGCAGAAAACA
Lgr5	CCTACTCGAAGACTTACCCAGT	GCATTGGGGTGAATGATAGCA
Lyz1	gccaaggtctacaatcgttgtgagttg	cagtcagccagcttgacaccacg
PLA2G2E	AGGATTCCCCCAAGGATGCCAC	CAGCCGTTTCTGACAGGAGTTCTGG
Reg3g	aacagaggtggatgggagtg	ggccttgaatttgcagacat
Tert	GCACTTTGGTTGCCCAATG	GCACGTTTCTCTCGTTGCG

Dataset 1. Source data for Figs 1-5

Click here to download Dataset 1
